# Activation-induced necroptosis contributes to B-cell lymphopenia in active systemic lupus erythematosus

**DOI:** 10.1038/cddis.2014.375

**Published:** 2014-09-11

**Authors:** H Fan, F Liu, G Dong, D Ren, Y Xu, J Dou, T Wang, L Sun, Y Hou

**Affiliations:** 1 The State Key Laboratory of Pharmaceutical Biotechnology, Division of Immunology, Medical School, Nanjing University, Nanjing, China; 2 Department of Immunology and Rheumatology, The Affiliated Drum Tower Hospital of Nanjing University Medical School, Nanjing, China; 3 Jiangsu Key Laboratory of Molecular Medicine, Nanjing, China

## Abstract

B-cell abnormality including excessive activation and lymphopenia is a central feature of systemic lupus erythematosus (SLE). Although activation threshold, auto-reaction and death of B cells can be affected by intrinsical and/or external signaling, the underlying mechanisms are unclear. Herein, we demonstrate that co-activation of Toll-like receptor 7 (TLR7) and B-cell receptor (BCR) pathways is a core event for the survival/dead states of B cells in SLE. We found that the mortalities of CD19^+^CD27^-^ and CD19^+^IgM^+^ B-cell subsets were increased in the peripheral blood mononuclear cells (PBMCs) of SLE patients. The gene microarray analysis of CD19^+^ B cells from active SLE patients showed that the differentially expressed genes were closely correlated to TLR7, BCR, apoptosis, necroptosis and immune pathways. We also found that co-activation of TLR7 and BCR could trigger normal B cells to take on SLE-like B-cell characters including the elevated viability, activation and proliferation in the first 3 days and necroptosis in the later days. Moreover, the necroptotic B cells exhibited mitochondrial dysfunction and hypoxia, along with the elevated expression of necroptosis-related genes, consistent with that in both SLE B-cell microarray and real-time PCR verification. Expectedly, pretreatment with the receptor-interacting protein kinase 1 (RIPK1) inhibitor Necrostatin-1, and not the apoptosis inhibitor zVAD, suppressed B-cell death. Importantly, B cells from additional SLE patients also significantly displayed high expression levels of necroptosis-related genes compared with those from healthy donors. These data indicate that co-activation of TLR7 and BCR pathways can promote B cells to hyperactivation and ultimately necroptosis. Our finding provides a new explanation on B-cell lymphopenia in active SLE patients. These data suggest that extrinsic factors may increase the intrinsical abnormality of B cells in SLE patients.

Systemic lupus erythematosus (SLE) is a typical autoimmune disease characterized by acute and chronic inflammation of the body, lymphopenia, a broad variety of autoantibodies and so on.^1^ Although the pathogenesis of SLE is still a puzzle,^2^ the abnormality of B cells is thought to be a central feature in SLE patients.^1, 3, 4^

The abnormality of B cells includes the decrease of absolute number,^5, 6, 7^ the altered frequency of their subsets^8, 9^ and hyperactivation and hyperresponsiveness to a variety of self-antigens and stimuli.^10, 11^ The defects of intrinsic signalings (such as Toll-like receptor 7 (TLR7) and B-cell receptor (BCR)) in B cells directly lead to lupus-like autoimmunity in mouse models,^12, 13, 14^ although the efficacy in clinical trials with B cell-depleting agents on SLE patients proved to be limited.^15, 16^ Moreover, gene expression microarrays can provide a wealth of molecular information for cells or tissues in different states. To date, only two papers involved in gene expression profiles of SLE B cells. One reported that there were 174 differentially expressed transcripts in active SLE B cells,^17^ whereas the other stated that 14 differentially expressed genes existed in quiescent SLE B cells,^18^ both of which provided a reference for the early onset of SLE. These studies suggest that extrinsic factors may induce abnormalities of B cells by acting on intrinsic signaling. In addition, it was reported that the anti-apoptotic cytokine signaling significantly influenced deregulation of cell death in SLE lymphocytes,^19^ but it is a pity that the differential gene expression profiles above did not fully reflect the survival status and immune function of active SLE B cells. Thus, it is still necessary to analyze the function states and gene expression profiles of B cells from SLE patients for understanding the underlying mechanism of the cell abnormality.

Interferon-*α* (IFN-*α*), a well-known cytokine in the pathogenesis of SLE, enhances the autoimmunity and immune response.^20, 21^ Both BCR and TLR7 signals play critical roles in autoreactive B cells contributing to autoimmune diseases.^22, 23, 24^ However, some reports indicated that repeated stimulation on TLR7 induced hyporesponsiveness (TLR tolerance) in B cells that was reversed by the activation of both BCR and IFN-*α* signals through the same PI3K/Akt/mTOR pathway.^25^ All above suggest that the intrinsic and extrinsic signals including IFN-*α*, TLR7 and BCR may be integrated together to positively regulate autoimmune and immune response of B cells. However, the effects of the integrated intrinsic and extrinsic signals on the survival status and immune function of B cells remain largely unknown.

In this study, we report that co-activation of TLR7 and BCR pathways promotes B cells to hyperactivation and ultimately necroptosis, suggesting that extrinsic factors may increase intrinsical abnormality of B cells in SLE patients. This study provides a new explanation on B-cell lymphopenia in active SLE patients.

## Results

### The elevated mortality of B cells in active SLE patients

**Figure 1:**
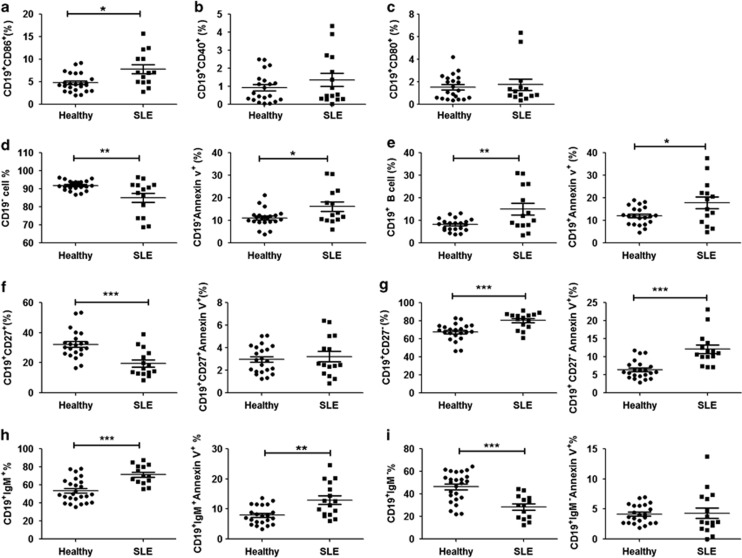
The elevated mortality of B cells in active SLE patients. Scatter plots represent the percentages of these B cell-subsets in 21 healthy controls (closed circles) and 14 SLE patients (closed squares). The mean of each set of values is shown as a horizontal line. (**a–c**) The percentage of CD86^+^ CD19^+^, CD80^+^CD19^+^ and CD40^+^CD19^+^ B cells. (**d**) The percentage of CD19^−^ cells and CD19^−^ Annexin V^+^ cells. (**e**) The percentage of CD19^+^ cells and CD19^+^Annexin V^+^ cells. (**f**) The percentage of CD27^+^CD19^+^ cells and CD27^+^ CD19^+^Annexin V^+^ cells. (**g**) The percentage of CD27^−^CD19^+^ cells and CD27^−^ CD19^+^ Annexin V^+^ cells. (**h**) The percentage of IgM^+^CD19^+^ cells and IgM^+^CD19^+^ Annexin V^+^ cells. (**i**) The percentage of IgM^−^CD19^+^ cells and IgM^−^ CD19^+^Annexin V^+^ cells. *P-*values (Students *t-*test) were calculated for the difference between SLE group and the control group. *P<0.05, **P<0.01 and ***P<0.001

A total of 14 active SLE patients and 21 healthy donors were recruited and peripheral blood mononuclear cells (PBMCs) were obtained. We first evaluated the activation status of SLE CD19^+^B cells in PBMCs. The results revealed that the activation marker CD86 was significantly upregulated in active SLE B cells compared with healthy donors (4.8±0.4% *versus* 7.8±1.0% Figure 1a), whereas the expression of CD40 and CD80 was unchanged (Figures 1b and c).

We next assessed the percentage of CD19^+^ B cells. Interestingly, both the proportion of CD19^+^ B cells in SLE lymphocytes (8.1±0.6% *versus* 15.0±2.6%) and the percentage of dead CD19^+^ B cells in total CD19^+^ B cells were increased (12.0±0.7% *versus* 17.8±2.6% ) compared with healthy donors (Figure 1e). Meanwhile, the proportion of CD19^−^ cells, mainly T cells, in SLE lymphocytes was decreased (91.88±0.5938% *versus* 85.05±2.618%) and the percentage of dead CD19^−^ cells in total CD19^−^ cells was increased (11.10±0.8412% *versus* 16.20±2.103% Figure 1d). Given T-cell apoptosis occurs in active SLE,^5, 26^ we speculate that abnormal homeostasis might also attribute to SLE B-cell apoptosis.

According to the cell surface marker CD27 or IgM, and cell death marker Annexin V, B-cell subpopulations were distinguished. The results showed that the proportion of CD19^+^CD27^+^ B cells (memory B cells) was reduced in active SLE patients (32.2±2.0% *versus* 19.6±2.3%), but their mortalities had no difference (3.0±0.3% *versus* 3.2±0.5%) compared with healthy donors (Figure 1f). The proportion of CD19^+^CD27^−^ B cells was increased significantly (67.8±2.0 *versus* 80.4±2.3) with elevated mortality (6.4±0.5% *versus* 12.1±1.2%) (Figure 1g). In addition, the proportion of CD19^+^IgM^+^ B cells (naive and mature B cells) was increased (53.63±2.603% *versus* 71.47±2.881%) with elevated mortality (7.9±0.6% *versus* 13.0±1.4%) (Figure 1h), whereas that of CD19+IgM^−^ B cells was decreased (46.4±2.6% *versus* 28.5±2.8%) without mortality difference (4.2±0.3% *versus* 4.3±0.9%) (Figure 1i).

Taken together, these data suggest that the elevated mortality of CD19^+^ B cell, mainly CD19^+^IgM^+^ and CD19^+^CD27^−^ B-cell subsets, may attribute to lymphopenia in active SLE patients.

### The gene expression profiles of active SLE B cells

**Figure 2:**
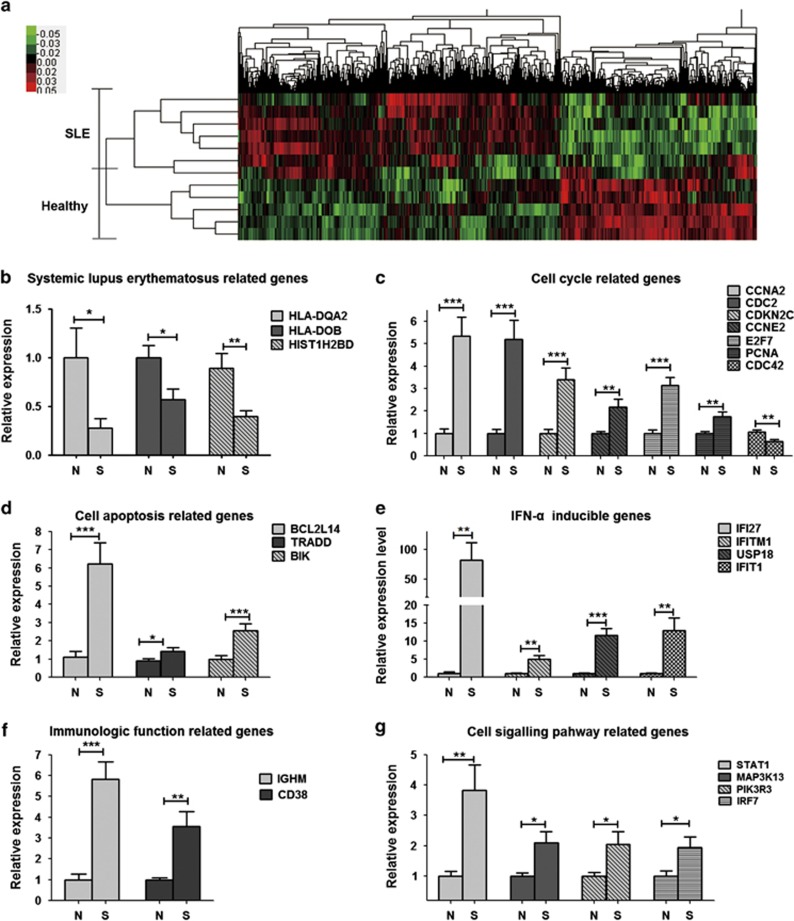
Hierarchical cluster dendrogram and verifications of SLE B-cell transcriptomes. (**a**) The differentially expressed genes are displayed significantly between CD19^+^ B cells isolated from the peripheral blood of SLE patients and healthy controls. The bars in the left indicate the associativity of SLE B-cell transcriptomes, and the bars on the top present clusters of differential expressed genes. Color changes indicate the expression level relative to the average (log2 scale). Red is increased expression, black is unchanged and green is decreased expression. The entire list of transcripts in the order as they appeared on the dendrogram is in Supplementary Table S1. (**b**–**g**) Selective verification of SLE B-cell differentially expressed genes. The mRNA was extracted from CD19^+^ B cells, and real-time PCR was performed. Bar graphs represent the fold change of mRNA levels in B cells between healthy controls (N) and SLE patients (S). The data are presented as relative mRNA levels following normalization. *P<0.05, **P<0.01 and ***P<0.001

To elucidate the molecular pathology feature of B cells in SLE, the differential gene expression profiles of CD19^+^ B cells between six active SLE patients and six healthy donors were assessed by gene microarrays. Hierarchical cluster analysis was performed for the changed genes with a ≥1.4-fold difference (*P*<0.05, Welch's *t*-test, BH-FDR). The results showed that 1017 genes were upregulated and 459 genes were downregulated in the active SLE B cells (Figure 2a).

To determine the most significant biological functions and pathways of the differentially expressed genes, the popular annotation databases KEGG and GO were applied and the results are shown in Supplementary Table S1 and Supplementary Figures S1–S4. Consistent with the previous reports, the signaling pathways including IFN, TLR, BCR, PI3K and MAPK and so on were involved. The results above proved that IFN, TLR and BCR signaling pathways were indeed activated in the B cells from active SLE patients.

### Validating the activated signals in active SLE B cells

Based on the clinical phenotypes and microarrays analysis of active SLE B cells, some differentially expressed genes related to SLE, apoptosis and cell cycle were selected and checked by real-time PCR. CD19^+^ B cells were separated from 50 ml PBMCs of 11 patients with active SLE and 11 healthy donors. The results showed that the expressions of genes related to SLE (*HLA-DQA2*, *HLA-DOB* and *HIST1H2BD*) were dramatically downregulated (Figure 2b), whereas the expressions of genes related to cell cycle (*CCNA2*, *CDC2*, *CDKN2C*, *CCNE2*, *E2F7* and *PCNA*) were significantly upregulated, except for *CDC42* (Figure 2c). In addition, the expressions of apoptosis-related genes (*BCL2L14*, *TRADD* and *BIK*) were also highly upregulated (Figure 2d).

The expressions of genes related to IFN-*α*, TLR7 and BCR signaling pathways were also verified. Indeed, the expressions of IFN pathway target genes (*IFI27*, *IFITM1*, *UPS18* and *IFIT1*) and the transcription factor *STAT1* were significantly upregulated (Figure 2e). Moreover, the expressions of genes related to B-cell immune function (*IGHM* and *CD38*) were significantly increased, whereas expression of MHC-inhibitory factor (*HLA-DOB*) was downregulated (Figure 2f). Of note, the expressions of downstream molecule of BCR and TLR7 pathway (*PIK3R3* and *MAP3K*13 kinases and transcription factor *IRF7*) were also significantly increased (Figure 2g).

These results proved that our microarray data are convincing and suggest that the high activation of IFN, BCR and TLR7 pathways may be involved in the elevated death of B cells from active SLE patients.

### Co-activation of IFN, TLR7 and BCR pathways induces normal B cells to achieve SLE-like status

**Figure 3:**
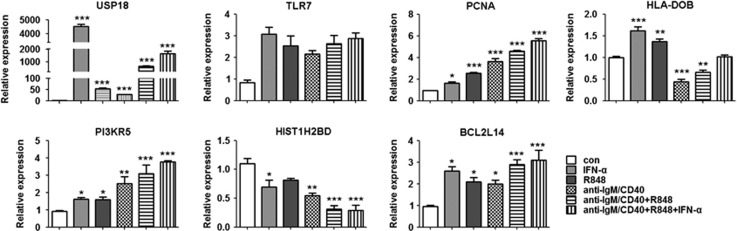
Co-activation of IFN, TLR7 and BCR pathways induces normal B cells to mimic the gene expression changes of SLE-like B cells. CD19^+^ B cells were isolated from the peripheral blood of healthy controls, and were stimulated with IFN-*α*, R848, anti-IgM/CD40, R848+anti-IgM/CD40 or IFN-*α*+R848+anti-IgM/CD40 for 6 h. After cells were harvested, mRNA was extracted and real-time PCR analysis was performed. Bar graphs represent the fold changes of USP18, TLR7, PCNA, HLA-DOB, PIK3R5, HIST1H2BD and BCL2L14 in mRNA levels of B cells with stimulations compared with the control. Data are presented as relative mRNA levels following normalization. *P<0.05, **P<0.01 and ***P<0.001

B cells from healthy donors were treated respectively with IFN-*α* or R848, a TLR7 ligand or anti-IgM/CD40 alone, or with two joint (anti-IgM/CD40+R848) or three joint (anti-IgM/CD40+R848+IFN-*α*) stimuli for 6 h, and then the expression of the selected target genes, based on above analysis on microarrays of active SLE B cells, was detected by real-time PCR. As expected, *USP18* and *TLR7* were upregulated with the treatment of IFN-*α*, representing the activation of IFN pathway. *TLR7* and *PCNA* were also upregulated with the treatment of R848, indicating the activation of TLR7 pathway. *HLA-DOB* was downregulated and *PIK3R5* was upregulated with the treatment of anti-IgM/CD40, indicating the activation of BCR pathway (Figure 3). Strikingly, three joint stimuli suppressed the expression levels of *HIST1H2BD* and *HLA-DOB*, but remarkably elevated the expression levels of *USP18*, *TLR7*, *PIK3R5*, *PCNA* and *BCL2L14* (Figure 3). These data indicated that the three joint stimuli made normal B cells, at least partially, to mimic the gene expression profile of SLE B cells.

**Figure 4:**
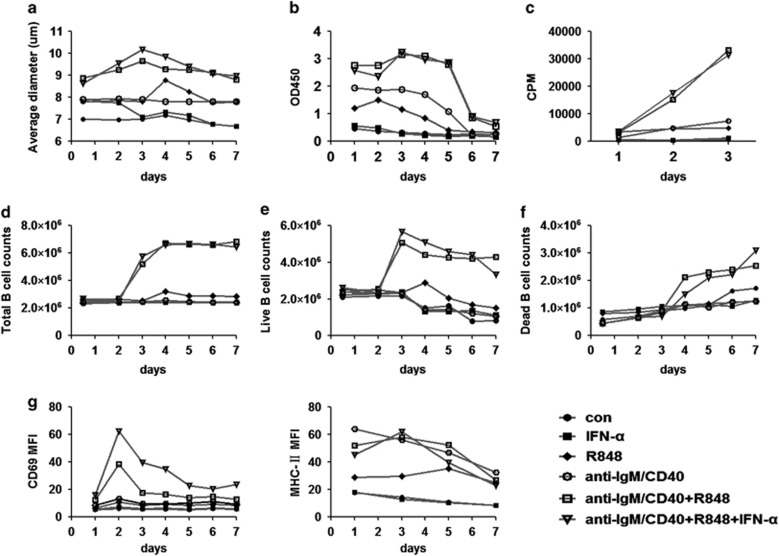
Co-activation of IFN, TLR7 and BCR pathways, even co-activation of TLR7 and BCR pathways, induces normal B cells to achieve SLE-like status. Mouse B cells isolated from spleen were cultured with the treatment of the separate or joint stimulation of IFN-*α*, R848 and anti-IgM/CD40 for 1 to 7 days and the index following were detected daily. Complete culture medium was changed every other day. The effects on the activation, proliferation and surface markers of B cells by separate or joint stimulation of IFN-*α*, R848, anti-IgM/CD40 were detected. (**a**) The average diameter dynamics of B cells. (**b**) The vitality dynamics of B cells was determined using Cell Counting Kit 8 assay kit and reading OD450 value. (**c**) The dynamics of H3 absorption. Stained by Trypan Blue, the total (**d**), live (**e**) and dead (**f**) B-cell numbers were counted by the cell counter. (**g**) Flow cytometry analysis of the MFI of CD69 and MHC-□. CPM, counts per min; MFI, mean fluorescence intensity

Next, we wondered whether co-activation of three pathways could cause changes in the physiological function of B cell from normal to SLE like. Although the responses of B cells to TLRs can vary between mice and humans, recent findings revealed that the IFN signaling regulates TLR7 similarly in mouse and human B cells.^25, 27^ Herein, B cells isolated from mouse spleen were treated with the indicated activators of IFN-*α*, TLR7 or BCR, and activation and death were detected during the following 7 days. As is shown in Figure 4, compared with stimulation of IFN-*α* or R848 or anti-IgM/CD40 alone, the two or three joint stimuli triggered B cells to acquire the biggest cell diameter (Figure 4a) and the strongest upregulation of CD69 and MHC-II expression (B-cell activation markers) (Figure 4g). In detail, only the joint stimulation enhanced the vitality and proliferation of B cells within the first 3 days (Figures 4b and c). Similarly, only the joint stimulation doubled the number of B cells on day 3 (Figure 4d), successively increased the number of dead B cells and decreased the number of the living cells from day 4 to day 7 (Figures 4e and f).

Taken together, these data indicated that joint stimuli, even co-activation of TLR7 and BCR pathways, induced normal B cells to achieve SLE-like status of the increased cell death along with overactivation.

### Co-activation of TLR7 and BCR pathways promotes necroptosis of B cells

Activation-induced cell death (AICD) usually means increased apoptosis after cell proliferation. However, we noticed that the average diameter of the joint stimulated B cells stubbornly remained at 9 *μ*m. As the reduced cell size is thought to be one feature of apoptosis, we suspect that apoptosis may not be the unique process of the B-cell death to explain lymphopenia in active SLE patients.

**Figure 5:**
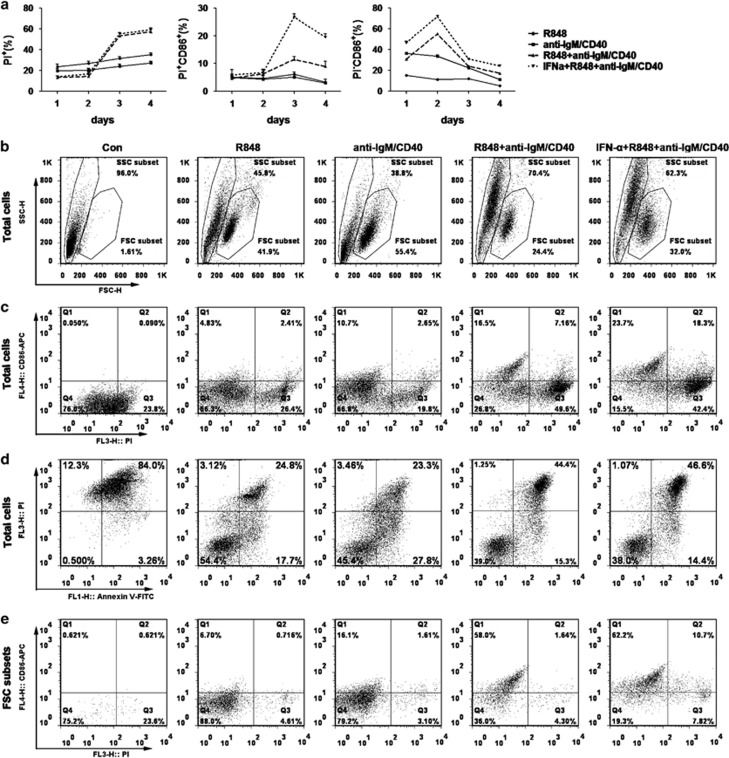
Co-activation of TLR7 and BCR pathways increases the mortality of B cells. Mouse spleen B cells were treated with R848, anti-IgM/CD40 or anti-IgM/CD40+R848 or R848+IFN-*α*+anti-IgM/CD40 for 4 days. Complete culture medium was changed every other day. (**a**) B cells were stained with PI and CD86-APC and cell death and activation were detected by flow cytometry. (**b**) The percentage of CD86^+^ PI^+^ cells in total B cells. (**c**) The percentage of Annexin V^+^ PI^+^ cells in total B cells. (**d**) The total B cells were distinguished as two subsets according to the forward scatter and side scatter parameters, and separately gated as FSC subsets and SSC subsets. FSC, forward scatter; SSC, side scatter. (**e**) The percentage of live (PI^−^) B cells in the FSC subset. Results are representative of three independent experiments

First, B cells were detected by flow cytometry with co-staining of CD86 and PI from day 1 to day 4. The results showed that joint stimulation provoked both the greatest mortality (PI^+^ %) and the highest PI^+^CD86^+^ rate of B cells compared with R848 or anti-IgM/CD40 alone (Figure 5a). Notably, IFN-*α* intensively strengthened the activation of B cells, but not significantly increased their mortality along with joint (R848+ anti-IgM/CD40) stimulations (Figures 5a and b). Furthermore, compared with R848 or anti-IgM/CD40 alone, the joint stimuli increased extremely not only the size and particle density of the B cells, but also the biggest percentage of the Annexin V^+^PI^+^ B cells (Figure 5c).

**Figure 6:**
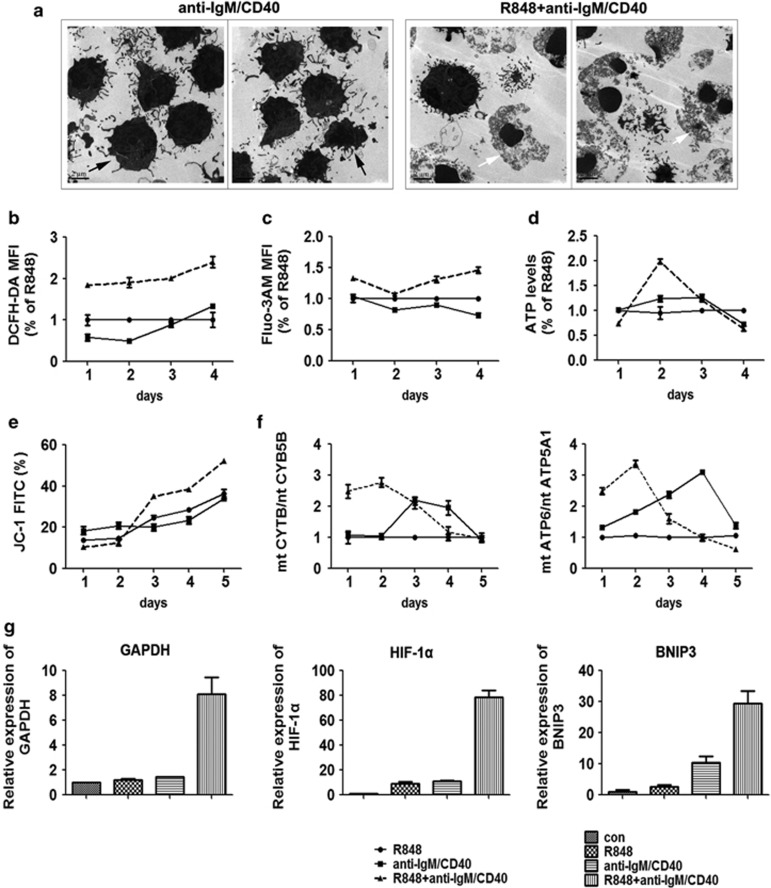
Co-activation of TLR7 and BCR pathways promotes the formation of necrotic ultrastructure, mitochondrial dysfunction and hypoxia of B cells. (**a**) The ultrastructure of mouse B cells treated with anti-IgM/CD40 or joint stimulation (R848+anti-IgM/CD40) for 4 days by transmission electron microscopy. Black arrowheads denote cell membrane integrity in the anti-IgM/CD40 treated cells. White arrowheads denote the swelling of cellular organelles and membrane breakdown in cells treated with joint stimulation. Among 200 cells counted, 92 showed necrotic morphology as presented in (**a**) in joint stimulated sample, whereas only 16 such cells were seen in anti-IgM/CD40-treated sample. (**b**) The mouse B cells stained with DCFH-DA for 4 days were detected by flow cytometry, and ROS production was assessed according to changes in the fluorescence intensity of DCF, the oxidation product of DCFH-DA. (**c**) The mouse B cells stained with Flou3-AM for 4 days were detected by flow cytometry, and Ca2^+^ influx was assessed according to changes in the fluorescence intensity of Flou3 and Ca2+combination. (**d**) The intracellular ATP levels were determined by the luciferase method and normalized by protein content. (**e**) Mitochondrial membrane potential (*ΔΨ*m) of mouse B cells in each treatment group was analyzed by JC-1 staining for 5 days. Loss of *ΔΨ*m was demonstrated by the percentage of JC-1 FITC. (**f**) Mitochondrial DNA quantification of mouse B cells using nuclear DNA (nDNA) as a reference was carried out by real-time PCR, and the results are presented as mtDNA/nDNA ratio. (**g**) The relative mRNA expression of target genes *GAPDH*, *HIF-1α* and *BNIP3* in mouse B cells with different stimulations were detected by real-time PCR on day 4, and *β*-tubulin was used as a reference. Mouse B cells without treatment as the control group were collected immediately after isolation from the spleen that were used to compare the target gene expression level

Next, we observed that activated B cells were significantly distinguished as two subgroups, named as FSC subset and SSC subset according to their size and particle density (Figure 5d). Meanwhile, B cells showed more percentage of SSC subset and less percentage of FSC subset with joint stimulation (Figure 5b). Interestingly, the FSC subset was almost PI^−^ cells (living or early apoptosis cells) (Figure 5e). At the same time, the morphology of B cells treated with anti-IgM/CD40 or R848+anti-IgM/CD40 was observed by transmission electron microscopy (TEM). The results showed that most of anti-IgM/CD40-treated B cells possessed normal cellular morphology including intact cytoplasmic membranes or apoptotic cellular morphology such as fragmented nuclei with condensed chromatin and formation of apoptotic bodies (Figure 6a), whereas most of R848+anti-IgM/CD40-treated B cells displayed necrotic cellular morphology, including electron-lucent cytoplasm, mitochondrial swelling and loss of plasma membrane integrity without severe damage to nuclei (Figure 6a). These data adequately indicated that co-activation of TLR7 and BCR pathways promoted activation-induced cell necroptosis (AICN) of B cells.

### B-cell necroptosis is accompanied by mitochondrial dysfunction and hypoxia

Recent research indicates that necroptosis was accompanied by reactive oxygen species (ROS) generation, calcium elevation, ATP depletion and mitochondrial dysfunction.^28, 29, 30, 31^ We observed that joint-stimulated B cells not only showed persistently strengthened ROS production and elevated calcium level in the cytoplasm from day 1 to day 4 (Figures 6b and c), but also had depletion of ATP production and loss of mitochondrial membrane potential (*ΔΨ*m) from day 3 (Figures 6d and e). Moreover, cell metabolism is often estimated by the ratio of mitochondrial DNA (mtDNA) to nuclear DNA (ntDNA).^32^ Mt-CYB (cytochrome *b*) and mt-ATP6 (ATP synthase 6) are encoded by mtDNA, whereas CYB5B and ATP5A1 are encoded by ntDNA. The ratio of mtDNA to ntDNA can be reflected by mtCYB/ntCYB5B or mtATP6/ntATP5A1.^33, 34^ We found that the ratio of mtCYB/ntCYB5B or mt ATP6/nt ATP5A1 was elevated in B cells treated with joint stimuli on the first 2 days, and subsequently decreased from day 3 (Figure 6f). These data indicated that the metabolism of mitochondria in B cells was seriously weakened after co-activation-initiated proliferation.

We also observed that the culture medium of B cells treated with joint stimuli became red in the first 2 days, and then turned to yellow from day 3. This phenomenon suggested increased glycolysis in activated B cells, a normal response to proliferation.^35^ B cells without treatment as the control group were collected immediately after isolation from the mouse spleen and used for analysis of the target gene or protein expression level. In fact, glyceraldehyde-3-phosphate dehydrogenase (GAPDH) is an energy metabolism-related critical enzyme in the glycolytic pathway. *GAPDH* was indeed significantly elevated in B cells treated with joint stimuli for 4 days (Figure 6g). Moreover, hypoxia-inducible factor-1*α* (HIF-1*α*) can target GAPDH.^36^ BNIP3 (BCL2/adenovirus E1B 19kD-interacting protein 3) is an atypical BH3-only member of the BCL-2 family of proteins and can be induced by HIF-1*α* and plays a critical role in cell apoptosis and necroptosis.^37^ Both *HIF-1α* and *BNIP3* were also significantly elevated in B cells treated with the joint stimuli (Figure 6g). These data indicated that B-cell necroptosis was also accompanied by hypoxia.

### Elevated expression of necroptosis-related genes in B cells of co-activation and from SLE patients

**Figure 7:**
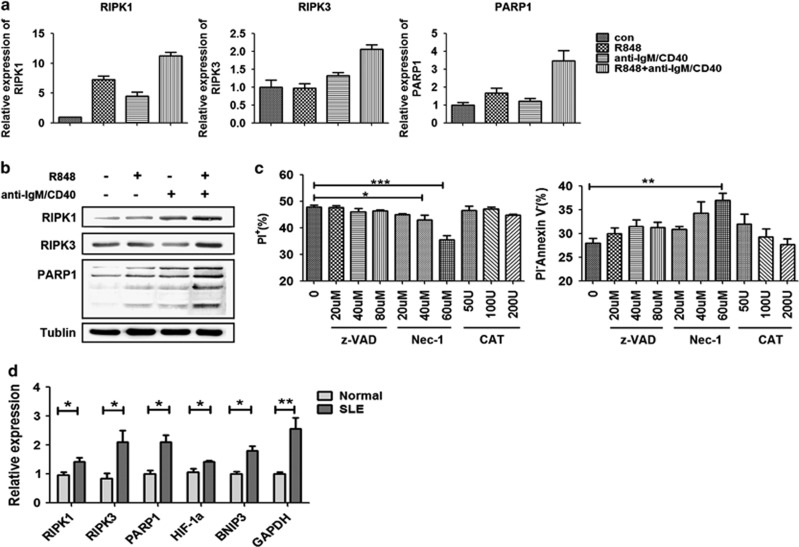
Elevated expressions of necroptosis-related genes are displayed in both the co-activation-induced B cells and the B cells from active SLE patients. (**a**) Mouse spleen B cells were exposed to the indicated stimulus and then harvested at day 4. The relative mRNA expression levels of necrotic-related genes *RIPK1*, *RIPK3* and *PARP1* were displayed in mouse spleen B cells. (**b**) Protein levels of RIPK1, RIPK3 and PARP1 were measured by western blot analysis in mouse spleen B cells. (**c**) Mouse spleen B cells were exposed to joint stimuli of R848 and anti-IgM/CD40 for 4 days after pretreatment with the pan-caspase inhibitor zVAD-fmk (20, 40 and 80 *μ*M), the necroptosis inhibitor Necrostatin-1 (Nec-1: 20, 40 and 60 *μ*M) and the ROS inhibitor Catalase (CAT: 50, 100 and 200 U) for 1 h. The B cells were co-stained with PI and Annexin V-FITC, and cell death was detected by flow cytometry. (**d**) The relative mRNA expression levels of necroptosis-related genes *RIPK1*, *RIPK3*, *PARP1*, *HIF-1α*, *BNIP3* and *GAPDH* were detected in B cells from eight SLE patients and eight healthy donors. Mouse B cells without treatment as the control group were collected immediately after isolation from the spleen, and were used to compare the target gene or protein expression level. *P<0.05, **P<0.01 and ***P<0.001

As is well known, RIPK1 (receptor-interacting protein kinase 1) and RIPK3 (receptor-interacting serine–threonine kinase 3) are critical regulators of necroptosis.^38, 39, 40^ PARP-1 (poly(ADP-ribose) polymerase-1), a DNA repair enzyme, is also involved in the induction of necroptosis.^41, 42^ We found that genes related to necroptosis, such as *RIPK1*, *RIPK3* and *PARP1*, were upregulated in B cells treated with joint stimuli at day 4, where *β-tubulin* was used as reference gene (Figure 7a). Accordingly, the protein expression levels of RIPK1, RIPK3 and PARP1 were also increased (Figure 7b).

Generally, necroptosis can be blocked by inhibition of RIPK1 kinase activity, and apoptosis can be rescued by a pan-caspase inhibitor zVAD-fmk. Catalase (CAT) can decompose ROS that is generated to cause cell death. Of note, pretreatment with Nec-1, a RIPK1 inhibitor, reduced the mortality of B cells (PI^+^) induced by joint stimuli and promoted their living cell rate (PI^−^Annexin V^−^), but B cells were not affected by zVAD-fmk or CAT (Figure 7c and Supplementary Figure S5).

To further verify whether B-cell lymphopenia in SLE is attributed to necroptosis, the expression levels of necroptosis-related genes were detected by B cells from eight SLE patients by real-time PCR, and *18S rRNA* was used as reference gene. Expectedly, the results showed that *RIPK1*, *RIPK3*, *PARP1*, *HIF-1α*, *BNIP3* and *GAPDH* were all significantly elevated in SLE B cells compared with those from healthy donors (Figure 7d).

Taken together, these data indicated that the mortality of B cells induced by joint stimuli and B-cell lymphopenia in active SLE patients may be mainly attributed to necroptosis.

## Discussion

SLE is associated with the dysregulation of B cells,^43^ mainly characterized by abnormal homeostasis and hyperactivation.^8, 9^ In this study, we first showed an increased mortality of CD19^+^CD27^−^ and CD19^+^IgM^+^ B-cell subsets from active SLE patients and a differential expression profile correlated to signaling pathways including IFN, TLR and BCR as well as the apoptosis, necroptosis and immune pathways.

Abnormal homeostasis and hyperactivation are the two important characteristics of SLE B cells.^2, 3^ Interestingly, CD27 expression has been considered as a universal memory B-cell marker. The reduced proportions (or the absolute number) of CD19^+^CD27^+^ B cells have already been considered as a main feature of active SLE B cells.^3, 4^ However, CD19^+^CD27^−^ B cells also represent a large fraction of memory B cells, and their percentage substantially increased in SLE patients.^4, 5, 6^ It was identified that marked B lymphopenia affected CD19^+^CD27^−^ B cells more than CD19^+^CD27^+^ B cells in SLE patients.^7^ Intriguingly, our results showed the proportion of CD19^+^CD27^−^ B-cell death was significantly increased, whereas the proportion of CD19^+^CD27^+^ B cells was indeed reduced in active SLE patient (Figures 1g and f). In addition, the proportion of CD19^+^IgM^+^ B-cell death was remarkably increased, whereas that of CD19^+^IgM^−^ B cells was decreased (Figures 1h and i). Thus, the death of CD19^+^CD27^−^ and IgM^+^CD19^+^ B cells may be involved in B lymphopenia.

B cells of active SLE patients do have unique gene expression profiles.^17, 44, 45^ Of note, our microarray results indeed showed that IFN, TLR and BCR pathways were all abnormally activated in active SLE B cells. It was reported that the agonists of BCR (anti-IgM plus anti-CD40) moderately promoted the proliferation of B cells.^46, 47^ Expectedly, joint stimulation of BCR with TLR7 triggered strongest proliferation in our present study (Figure 4d). In addition, our results also showed that IFN-*α* promoted the two joint (BCR plus TLR7) stimuli-induced activation to the highest degree. We thus suppose that overactivation of IFN-*α*, TLR7 and BCR pathways could enough induce dysregulation of B cells in active SLE patients.

The joint stimuli promoted AICN of B cells. Interestingly, we found that joint stimulation provoked both the greatest mortality (PI^+^ %) and the highest PI^+^CD86^+^ rate of B cells (Figure 5a). The reduced size is thought to be one feature of apoptotic cell, and joint stimulation increased the percentage of SSC subset of B cells (Figure 5b) and displayed necrotic cellular morphology (Figure 6a). Furthermore, recent research indicates that necroptosis was accompanied by ROS generation, calcium elevation, ATP depletion and mitochondrial dysfunction^28, 29, 30, 31^ that also happened to necroptotic B cells. We also observed that the culture medium of B cells with joint stimuli turned to yellow, which means a decrease in pH, that may be caused by hypoxia in anaerobic glycolysis.^35^ HIF-1*α* regulates the expression of all enzymes in the glycolytic pathway.^48^ The intermediary metabolites of the glycolytic pathway are essential for cell growth and the maintenance of cells.^49, 50^ In our present study, the activated B cells exhibited significantly elevated expression of HIF-1*α* and its target gene GAPDH. Hence, we identified that joint stimuli may promote hypoxia of B cells.

Recent studies reported that necroptosis is dependent on the kinases RIPK1 and RIPK3.^51^ PARP-1 is also involved in the induction of necroptosis.^41, 42^ We also found that RIPK1, RIPK3 and PARP1 were upregulated in B cells treated with the joint stimuli (Figures 7a and b). Generally, the inhibition of RIPK1 kinase activity is commonly used to estimate necroptosis. Apoptosis is blocked by a pan-caspase inhibitor zVAD-fmk. Catalase can decompose ROS that is generated to cause cell death. It was reported that microglia activated through TLR1/2,3,4,7/8 undergo RIP1/RIP3-dependent necroptosis when exposed to the pan-caspase inhibitor zVAD-fmk that was completely blocked by R1PK1 kinase inhibitor necrostatin-1.^38^ Ripk1-deficient mice die at birth from systemic inflammation.^52^ In addition, the BCR pathway also induces the resting B cells to enter into cell cycle, proliferation and differentiation or AICD.^51^ We found that pretreatment with Nec-1 reduced the mortality of B cells induced by joint stimuli, but the B cells were not affected by zVAD-fmk or CAT (Figure 7c). Expectedly, genes related to necroptosis including RIPK1, RIPK3 and PARP1 were also significantly elevated in SLE B cells compared with those from healthy donors (Figure 7d). In addition, it is already known that necroptotic cell lysates account for increased inflammation and antinuclear antibody production, whereas apoptotic death occurs without exciting an inflammatory response.^53^ This also suggests to us that necroptosis may be the main manner of death in active SLE B cells.

In conclusion, we demonstrate that co-activation of TLR7 and BCR pathways is a core event for the survival/dead states of B cells and also promotes B cells to hyperactivation and ultimately necroptosis. Our finding provides a new explanation on B-cell lymphopenia in active SLE patients. These data suggest that extrinsic factors may increase the intrinsical abnormality of B cells in SLE patients.

## Materials and Methods

### Study participants

The study protocol was approved by the research ethics committee of Nanjing University. Whole blood was obtained with written informed consent from each patient and healthy subject. All SLE patients were diagnosed according to the criteria set out by American College of Rheumatology revised criteria in 1997. Disease activity was evaluated using the SLE Disease Activity Index (SLEDAI) with a cutoff of ≥8 that was used to define active disease. For flow cytometric analysis, 2 ml whole blood of 21 healthy subjects (8 males and 13 females) with a mean (±S.D.) age of 25±6 years and 14 SLE patients (4 males and 10 females) with a mean (±S.D.) age of 28±7 years were recruited. For the microarray, 6 healthy subjects (3 males and 3 females) from local blood bank (50 ml) with a mean (±S.D.) age of 27±6 years were recruited. Six patients with active SLE patients (3 males and 3 females), who were hospitalized at the clinical unit of the Affiliated Drum Tower Hospital of Nanjing University Medical School, with a mean (±S.D.) age of 30±7 years, were recruited for whole blood (50 ml). For real-time PCR verification, 50 ml whole blood of 11 healthy subjects (5 males and 6 females) with a mean (±S.D.) age of 28±7 years and 11 SLE patients (4 males and 7 females) with a mean (±S.D.) age of 30±9 years were recruited. For necroptotic analysis, 50 ml whole blood of 8 healthy subjects (2 males and 6 females) with a mean (±S.D.) age of 24±5 years and 8 SLE patients (2 males and 6 females) with a mean (±S.D.) age of 27±6 years were recruited. Although the patients were on a variety of disease-modified agents, those on high-dose immunocytotoxic therapies or steroids were excluded from the study.

### Animals

Female C57BL/6 mice, 6–8 weeks old, were purchased from Model Animal Research Center of Nanjing University (Nanjing, China). The animals were fed a diet of standard rodent chow and water. All mice were acclimated to the surrounding environment for ≥1 week before use. The experiments were conducted according to institutional animal ethics guidelines.

### Isolation, culture and treatment of human and mouse B cells

PBMCs were obtained by Ficoll density gradient centrifugation. Human B cells were purified using a human CD19 B Cell Isolation Beads (Miltenyi Biotec, Bergisch Gladbach, Germany) according to the recommendations of the manufacturer. Mouse spleen B cells were purified using a mouse CD45R (B220) beads according to the recommendations of the manufacturer.

### B-cell culture and treatment

Human or mouse B cells were cultured in 96-well flat-bottom plates (Corning, Tewksbury, MA, USA) at 5 × 10^5^ cells per well in 200 *μ*l RPMI-1640 (Gibco, Grand Island, NY, USA) supplemented with 10% FBS (Gibco) and antibiotics (penicillin 100 *μ*g/ml, streptomycin 10 *μ*g/ml; Invitrogen Life Sciences, Carlsbad, CA, USA) in a humidified atmosphere of 5% CO_2_ at 37°C. For stimulation treatment, B cells were divided into four to six groups: Control (grown in normal media), IFN-*α* (1000 U/ml, eBiosciences, San Diego, CA, USA), R848 (1 *μ*g/ml, Enzo Life Science, Farmingdale, NY, USA), affiniPure F(ab')2 Fragment Goat Anti-Mouse IgM (5 *μ*g/ml, Jackson ImmunoResearch Laboratories, Inc., West Grove, PA, USA) plus anti-CD40 (2 *μ*g/ml, eBiosciences), two joint stimuli (anti-IgM/CD40+R848) and three joint stimuli (anti-IgM/CD40+R848+IFN-*α*). Each group of B cells was stimulated respectively on the first day, and the culture medium was changed every other day. Mouse B cells without treatment as the control group were collected immediately after isolation from the spleen, and used to compare the target gene or protein expression level. In addition, mouse B cells were exposed to the stimuli after pretreated with inhibitor zVAD-fmk (Merck, Billerica, MA, USA), Necrostatin-1 (Merck) or Catalase (Beyotime, Nantong, China) for 1 h.

### H3 detection

For ^3^H-Thymidine uptake, human B cells were cultured with their respective stimuli in 96-well plates at a density of 2.5 × 10^6^ cells per ml. B cells were pulse-labeled with [^3^H]thymidine (0.2 *μ*Ci per well, Amersham Life Science, Little Chalfont, UK) for 6 h before harvest. Cells were harvested over glass fiber filters and radionuclide uptake was measured by scintillation counting. All experiments were performed in triplicate and repeated three times.

### Cell counting

Dilute B-cell sample in Trypan Blue by preparing a 1 : 1 dilution of the cell suspension using 0.4% Trypan Blue solution. Nonviable cells will be blue, and viable cells will be unstained. The total cells, live cells or dead cells were automatic counted by Countstar automated cell counter (Biomen Biomen Biosystems CO., LTD, Guangzhou, China).

### RNA isolation and microarray

The purified human B cells were centrifuged in RNase-free tubes treated with Trizol Reagent (Invitrogen, Carlsbad, CA, USA). Total RNA was extracted by using the RNeasy kit according to the instructions of the manufacture (Qiagen, Valencia, CA, USA) and then RNA concentration was assessed by Nanodrop 2000 spectrophotometry (Thermo, Waltham, MA, USA). RNA quality was determined by formaldehyde denaturation electrophoresis and only those samples with a 260 nm/280 nm optical density ratio (OD260/280) >1.8 and a total RNA concentration >1 mg/ml were submitted for hybridization to generate labeled targets. Then, the total RNA was used to synthesize double-strand cDNA (ds-cDNA) using an Invitrogen SuperScript ds-cDNA synthesis kit in the presence of oligo dT primers. The ds-cDNA was cleaned and labeled using NimbleGen One-Color DNA Labeling Kit in accordance with the manufacturer's protocol (NimbleGen Systems, Inc., Madison, WI, USA). The labeled ds-cDNA was hybridized with NimbleGen 12 × 135 K Human Gene Expression Microarray consisting of 45 033 probes for human genes (Hybridization System-NimbleGen Sys-199 tems, Inc.). Following hybridization, washing was performed using the NimbleGen Wash Buffer kit (NimbleGen Systems, 201 Inc.). After being washed in an ozone-free environment, the processed slides were scanned with the Roche-NimbleGen MS200 confocal laser scanner (CapitalBio Corporation, Beijing, China) using the recommended settings.

### Analysis of microarray data

The obtained images were analyzed using NimbleScan software (version 2.5) for grid alignment and expression data analysis. Expression data were normalized through quantile normalization and the Robust Multichip Average (RMA) algorithm. All gene-level files were imported into Agilent GeneSpring GX software (version 11.5.1; Agilent, Santa Clara, CA, USA) for further analysis. Genes showing minimal variation between defined phenotypes were excluded from analysis using a fold change filter set at 1.4-fold. Hierarchical clustering and principal components analysis using an uncentered correlation distance metric and average linkage clustering were performed in Cluster^54^ (cluster bb3.0, http://rana.lbl.gov/eisen/) with visualization in Treeview^55^ (treeview 1.60 programs, http://rana.lbl.gov/eisen/). To evaluate the potential biological significance of the changes observed in the microarrays, network analysis of the differentially expressed genes were performed, using by the CapitalBio-Molecule Annotation System (MAS) software (http://bioinfo.capitalbio.com/mas3). To determine the most significant biological functions and pathways of the differentially expressed genes, the two popular annotation databases, GO and Kyoto Encyclopedia of Genes and Genomes (KEGG), were applied.

### Quantitative real-time PCR analysis

Real-time PCR for the verification of differential genes in the paper was performed on an Applied Biosystems StepOne Sequence Detection System (Applied Biosystems, Foster city, CA, USA). TaqMan primers and probe of targeted homo genes were designed with the Roche universal probe library (Roche Applied Science, Penzberg, Germany). The Roche TaqMan probes were labeled at the 5′ end with the FAM reporter dye and were labeled at the 3′ end with the quencher dye BHQ1 (Roche Molecular Biochemicals, Indianapolis, IN, USA). For quantification, the relative mRNA level of specific gene expression was obtained using the 2^-ΔΔCt^ method.

### Flow cytometric analysis

Monoclonal antibodies against human CD19, CD86, CD80, CD40, IgM, CD27 and isotype-matched controls, as well as monoclonal antibodies against mouse CD69, CD138, IgM, MHC-II and isotype-matched controls, were obtained from eBiosciences. Labeled cells were analyzed on FACS Calibur flow cytometer BD Biosciences (San Diego, CA, USA) and data were analyzed using FlowJo software (Treestar, Inc., San Carlos, CA, USA).

### Cell viability analysis

Cell viability was assessed with a Cell Counting Kit 8 (Dojindo Laboratories Inc., Mashikimachi, kamimashiki gun, Japan), according to the recommendations of the manufacturer.

### Cell apoptosis assay

Apoptosis was assayed with an Annexin V-FITC and PI Apoptosis kit, according to the recommendations of the manufacturer (eBiosciences).

### Transmission electron microscopy

Samples were prepared for TEM by overnight fixation at 4°C in 2% glutaraldehyde/0.1 M cacodylate (pH 7.4). Randomly chosen fields were viewed with a Jeol 1200EX Biosystem TEM (JOEL, Peabody, MA, USA).

### Measurement of Intracellular ATP

The cellular ATP content was determined using a bioluminescence assay according to the manufacturer's instructions (Beyotime) by the luciferase reporter assay system (Promega, Madison, WI, USA). Each data point was normalized to the cell protein contents.

### Measurement of ROS, Ca^2+^ and *ΔΨ*m

DCFH-DA was used to detect ROS production, Flou3-AM was used to detect Ca^2+^ influx and JC-1 was used to detect *ΔΨ*m according to the recommendations of the manufacturer (Beyotime).

### Western blot

The method of western blotting was performed as previously described. RIPK1 and *β*-tubulin were from Cell Signaling Technology (Danvers, MA, USA). RIPK3 was from GeneTex (Irvine, CA, USA). Parp1 (H-25) was from Santa Cruz (Dallas, TX, USA).

### Statistical analysis

Results were presented as mean±S.E.M. Student's *t*-test was used to compare between two groups. Differences were considered to be significant with *P-*values of <0.05. Statistical calculations were performed with GraphPad Prism software (San Diego, CA, USA).

## Abbreviations

SLE: systemic lupus erythematosus
PBMC: peripheral blood mononuclear cell
TLR7: Toll-like receptor 7
BCR: B-cell receptor
IFN-*α*: interferon-*α*
AICD: activation-induced cell death
AICN: activation-induced cell necroptosis
TEM: transmission electron microscopy
*ΔΨ*m: mitochondrial membrane potential
ROS: reactive oxygen species
mtDNA: mitochondrial DNA
ntDNA: nuclear DNA
mt-CYB: cytochrome *b*
mt-ATP6: ATP synthase 6
CYB5B: cytochrome *B*5 outer mitochondrial membrane
ATP5A1: mitochondrial ATP synthase
HIF-1*α*: hypoxia-inducible factor-1*α*
GAPDH: glyceraldehyde-3-phosphate dehydrogenase
BNIP3: BCL2/adenovirus E1B 19kD-interacting protein 3
PARP-1: poly(ADP-ribose) polymerase-1
RIPK1: receptor-interacting protein kinase 1
RIPK3: receptor-interacting serine–threonine kinase 3
CAT: catalase
