# Supplementary material for: Activation-induced necroptosis contributes to B-cell lymphopenia in active systemic lupus erythematosus
Source: Cell Death Dis. 2014 Sep 11;5(9):e1416–. doi: 10.1038/cddis.2014.375 (PMC4225223; doi:10.1038/cddis.2014.375)
Supplement: Supplementary Information [file cddis2014375x1.doc]

**Supplementary information**

**II. Supplementary result**

**1. Genes related to IFN, TLR and BCR pathways are up-regulated in active SLE B cells**

IFN, TLR and BCR pathways are all abnormally activated in SLE B cells, which were reflected in our microarray results. The components of IFN-JAK-STAT signaling pathway (*IFNAR* 1, *STAT* 1 and *STAT* 2) as well as the target genes of IFN (*IFI* 27, *IFI* 6 and *IFITM* 1) were all up-regulated. *TLR* 7 and transcription factor *IRF* 7 of TLR pathway were also significantly up-regulated. Moreover, the BCR component *IGHM*, and its down-stream tyrosine kinase *BLK* and PIK 3 kinase family member (including *PIK3CG*, *PIK3R* 3, *PIK3R* 5 and *PIK3AP* 1) were also significantly increased. Furthermore, MAPK kinases *MAP2K* 6, *MAPK* 13 (*P* 38), *MAP4K* 3 and *MAP3K* 13, which are the down-stream kinases of both TLR and BCR pathways, were also significantly increased (Table S1). In addition, B cell functions as antigen presentation. Our microarrays analysis showed the genes including the antigen presentation negative regulatory factor *HLA-DOB*, the MHC-Ⅱ molecule component *HLA-DQA* 2, and nucleosomal histone *H2A, H2B, H* 3 and *H* 4 were down-regulated, while the positive regulators *BLK* and *STAP* 2 were up-regulated, indicating that B cell is indeed over-activated in active SLE patients(Table S1). These results suggest that the function status of B cells in active SLE patients were abnormally activated.

**2. Genes related to apoptosis and cell cycle are changed in active SLE B cells**

The low absolute number of B cells in active SLE patients is clinical indicator and high apoptosis rate was observed in B cells from Chinese active SLE patients (Figure 1). Accordingly, our microarray analysis also showed the apoptotic-related genes (*BCL2L* 14, *BIRC4BP* and *BIK*) were significantly up-regulated, while anti-apoptotic genes (*BAG* 3 and *BAG* 4) were down-regulated. However, some genes related B cell activation and proliferation *TNFSF13B* (*BAFF*) and *TNFRSF* 17 as well as anti-apoptotic genes (*BIRC* 5 and *BCL2L* 10) were significantly up-regulated, while the apoptotic genes (*TNFRSF10B* and *CYCS)* were down-regulated (Table S1). On the other hand, the expression of many important genes related to cell cycle were also changed, for example, the G1/S transition-related genes (*CDC25A*, *CDC* 6 and *CCND* 3*)*, the blocking cell cycle-related genes (*WEE* 1, *CHEK* 1, *E2F* 6 and *CDKN2A*), and DNA replication-related genes (*MCM* 10, *MCM* 4 and *PCNA)* were all up-regulated (Table S1). These data indicate the survival status of SLE B cells is indeed closely related to the abnormal homeostasis of their apoptosis and cell cycle. In addition, our SLE B cell microarrays also showed that genes related to SLE, such as *CD* 86, *C1QB* and *C1QC,* were up-regulated. Meanwhile, we also found some interesting changes of unreported genes, for example, the down-regulation of the integral histone family genes (23 genes in total including the subtypes of *H2A*, *H2B*, *H* 3 and *H* 4).

Table S1 A comprehensive list of the transcripts, fold change and molecular functions for active SLE B cells compared to healthy donors B cells


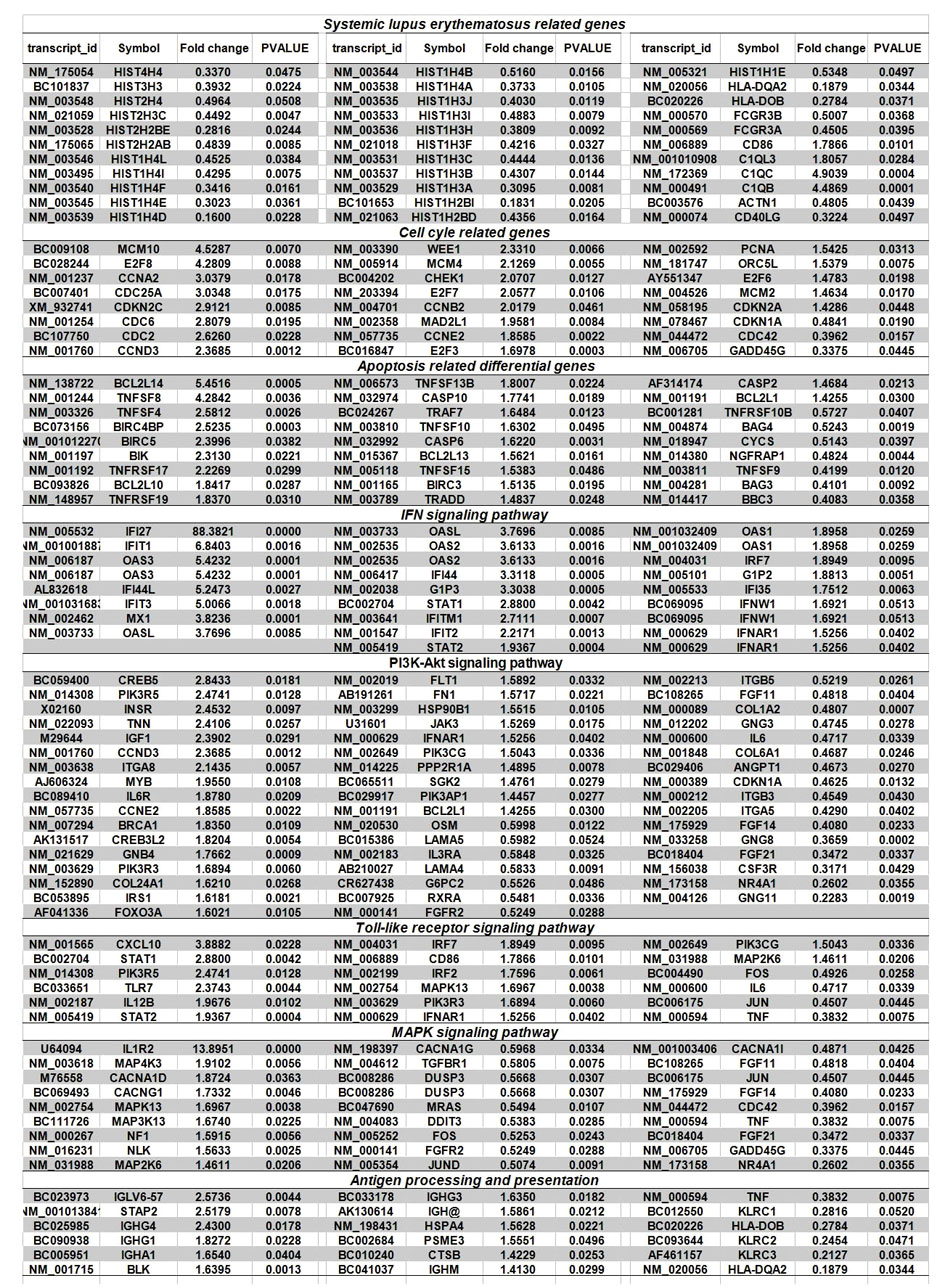

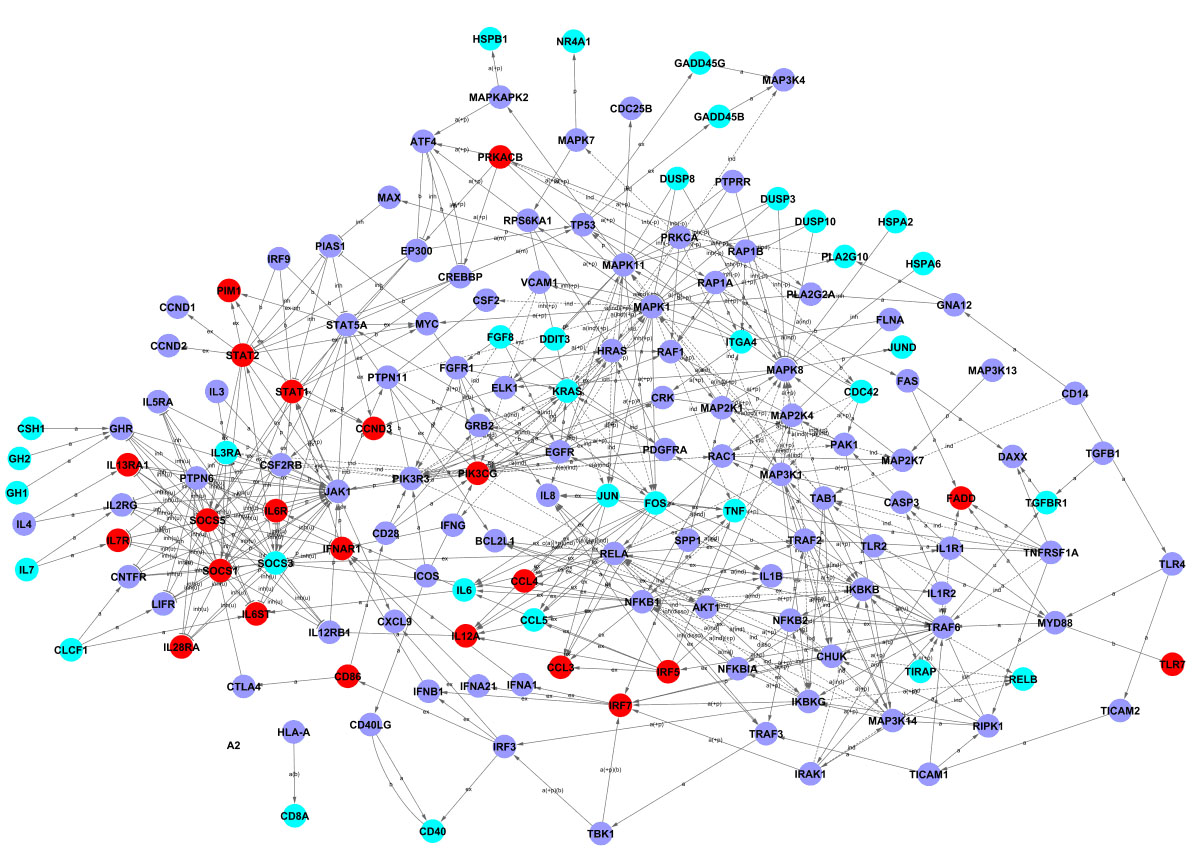


Figure S1. The first analysis of signal network map in active SLE B cell. The signal network was mapped according to the differential expression genes of CD19^+^ B cells from active SLE patients compared to healthy control. The pathways, including Jak-STAT signaling, Toll-like receptor (TLR) signaling, MAPK signaling and cell adhesion molecules (CAMs) signaling, were changed. Meanwhile, the components of IFN-JAK-STAT signaling pathway (*IFNAR* 1, *STAT* 1 and *STAT* 2) and *TLR* 7 and transcription factor *IRF* 7 of TLR pathway were significantly up-regulated. The target genes of IFN (*IFI* 27, *IFI* 6 and *IFITM* 1 etc) were also all up-regulated. The detail data of gene expression was provided in the Table S1. Blue, down-regulated genes in the signal network; Red, up-regulated genes in the signal network.


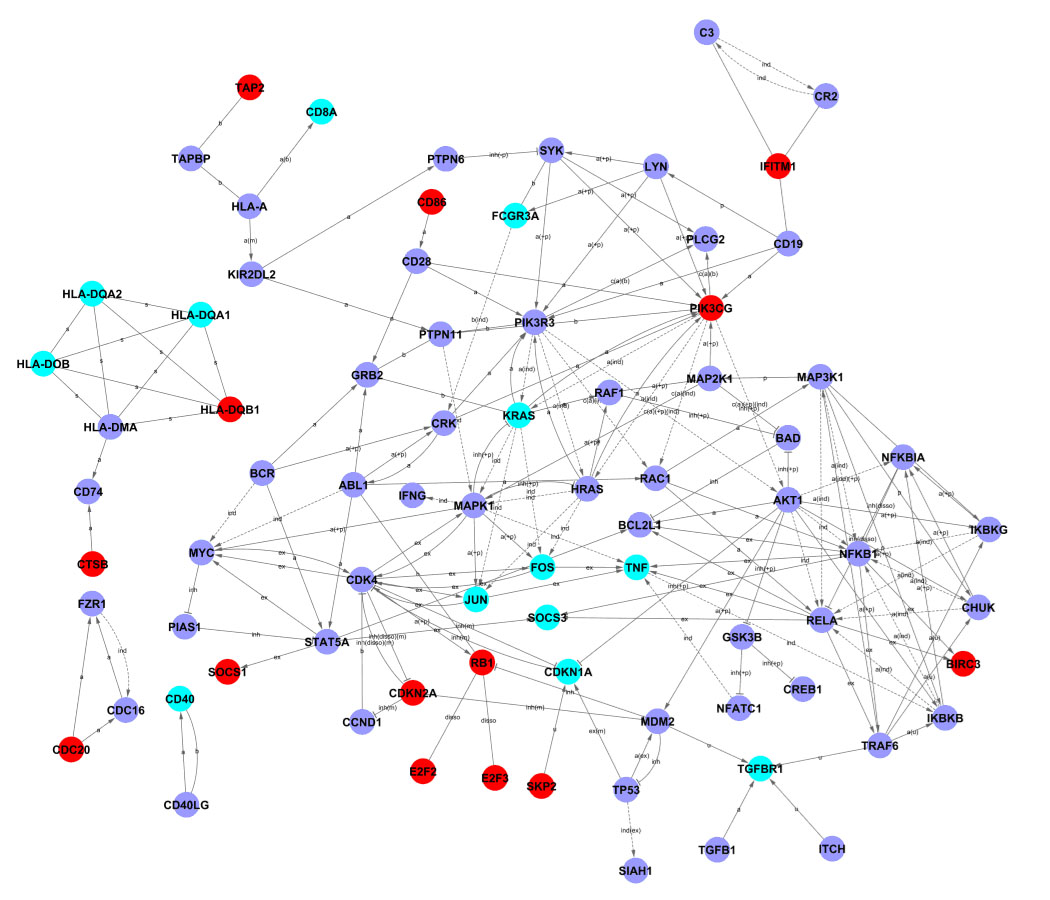


Figure S2. The second analysis of signal network map in active SLE B cell. Signal network maps revealed that pathway of B cell immunologic function, such as systemic lupus erythematosus, B cell receptor (BCR) signaling pathway, antigen processing and presentation and ubiquitin mediated proteolysis, were involved in the changes of B cells in SLE patients. PIK 3 kinase family member PIK3CG located the down-stream of BCR was significantly increased. BCR component IGHM, its down-stream tyrosine kinase BLK and PIK 3 kinase family member (including PIK3CG, PIK3R 3 and PIK3R 5, PIK3AP 1), and MAPK kinases (MAP2K 6, MAPK 13, MAP4K 3 and MAP3K 13) were also significantly increased and detail data on gene expression provided in the Table S1. Blue, down-regulated genes in the signal network; Red, up-regulated genes in the signal network.


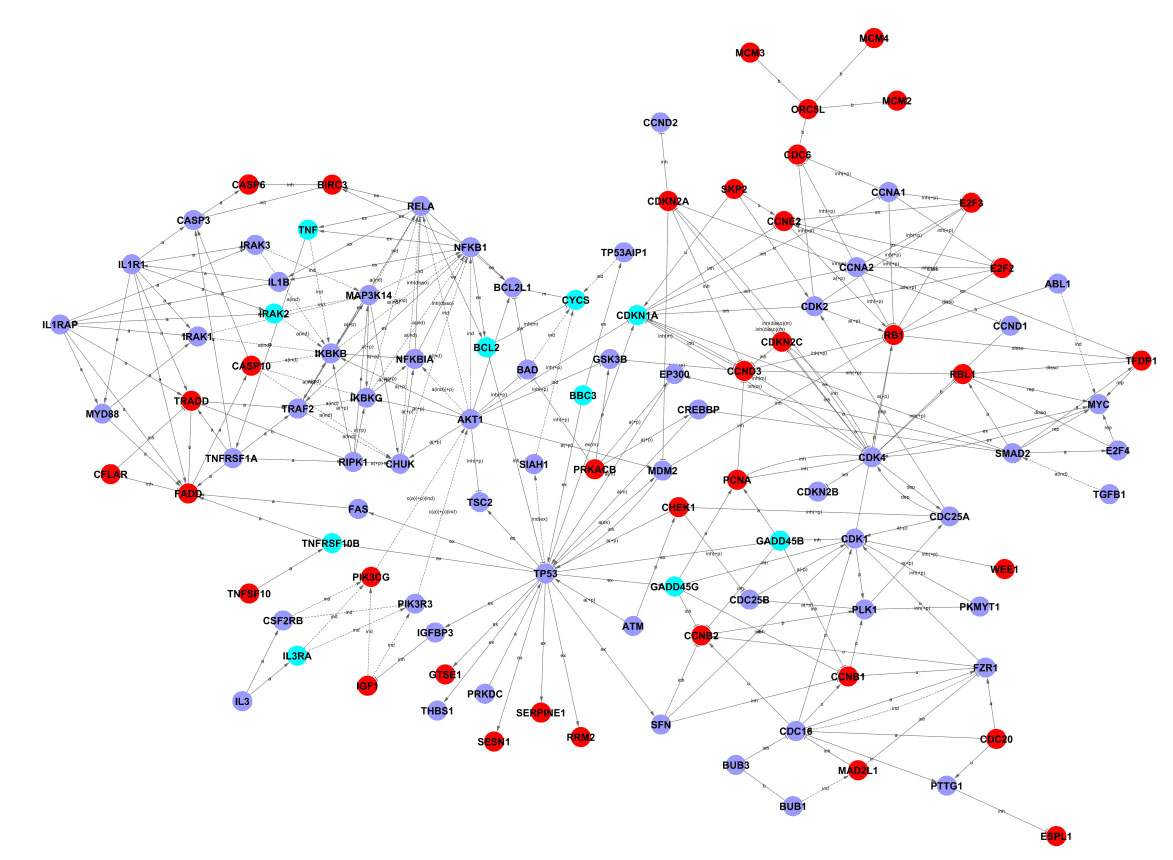


Figure S3. The third analysis of signal network map in active SLE B cell. Signal network maps, which were constructed by outlining the interactions of genes, revealed that pathways of cell cycle and cell apoptosis were invovled in the changes of B cells in SLE patients. The apoptotic genes (TNFRSF10B and CYCS) were down-regulated, while the genes related to cell cycle (CDC 6 and CCND 3, WEE 1, CHEK 1, CDKN2A, MCM 4 and PCNA) were all up-regulated and detail data on gene expression provided in the Table S1. Blue, down-regulated genes in the signal network; Red, up-regulated genes in the signal network.


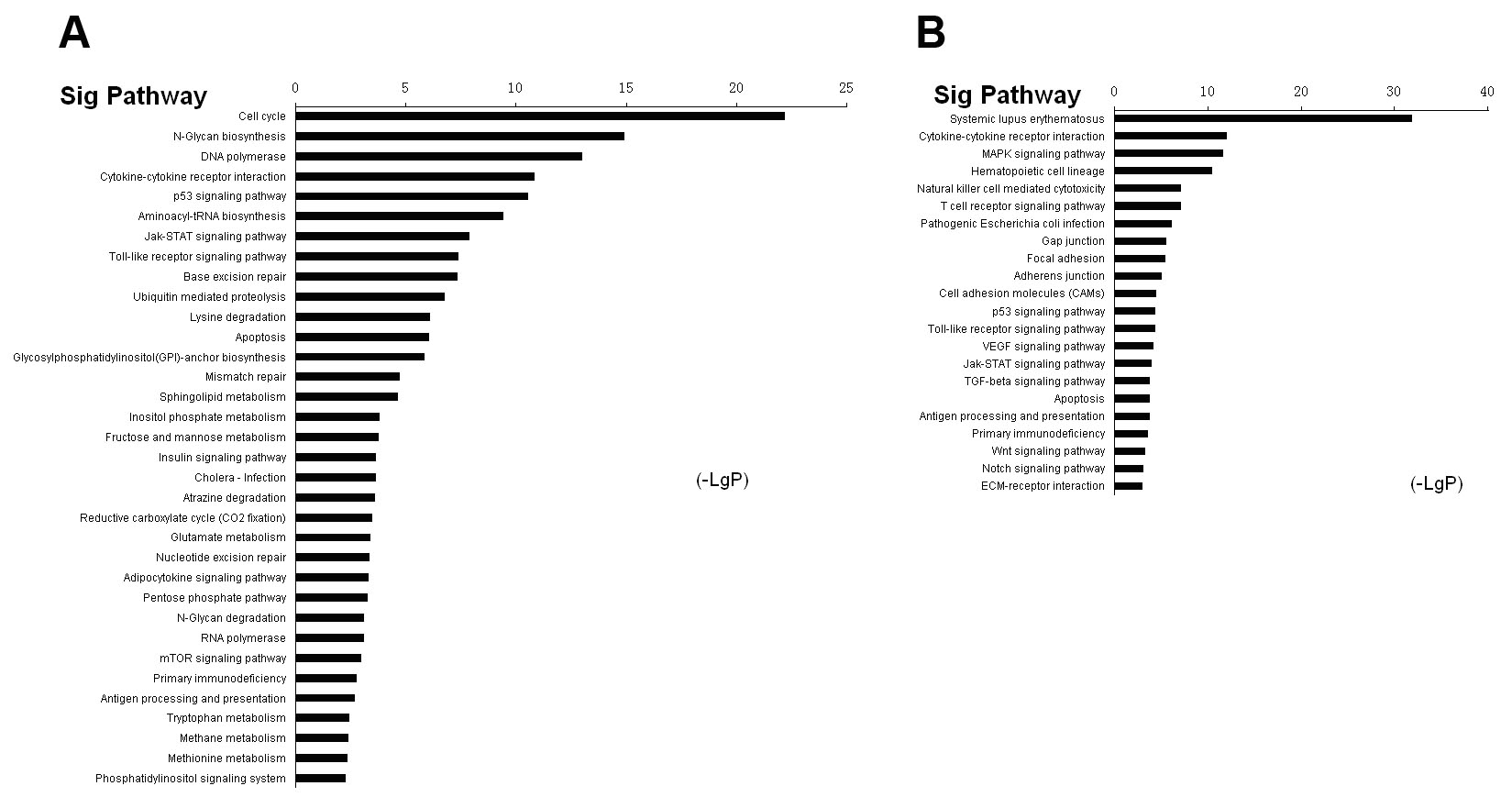


Figure S4. The classification analysis of the differential expression genes in active SLE B cell. （**A**）34 up-regulated and (**B**) 22 down-regulated signal transduction pathways.


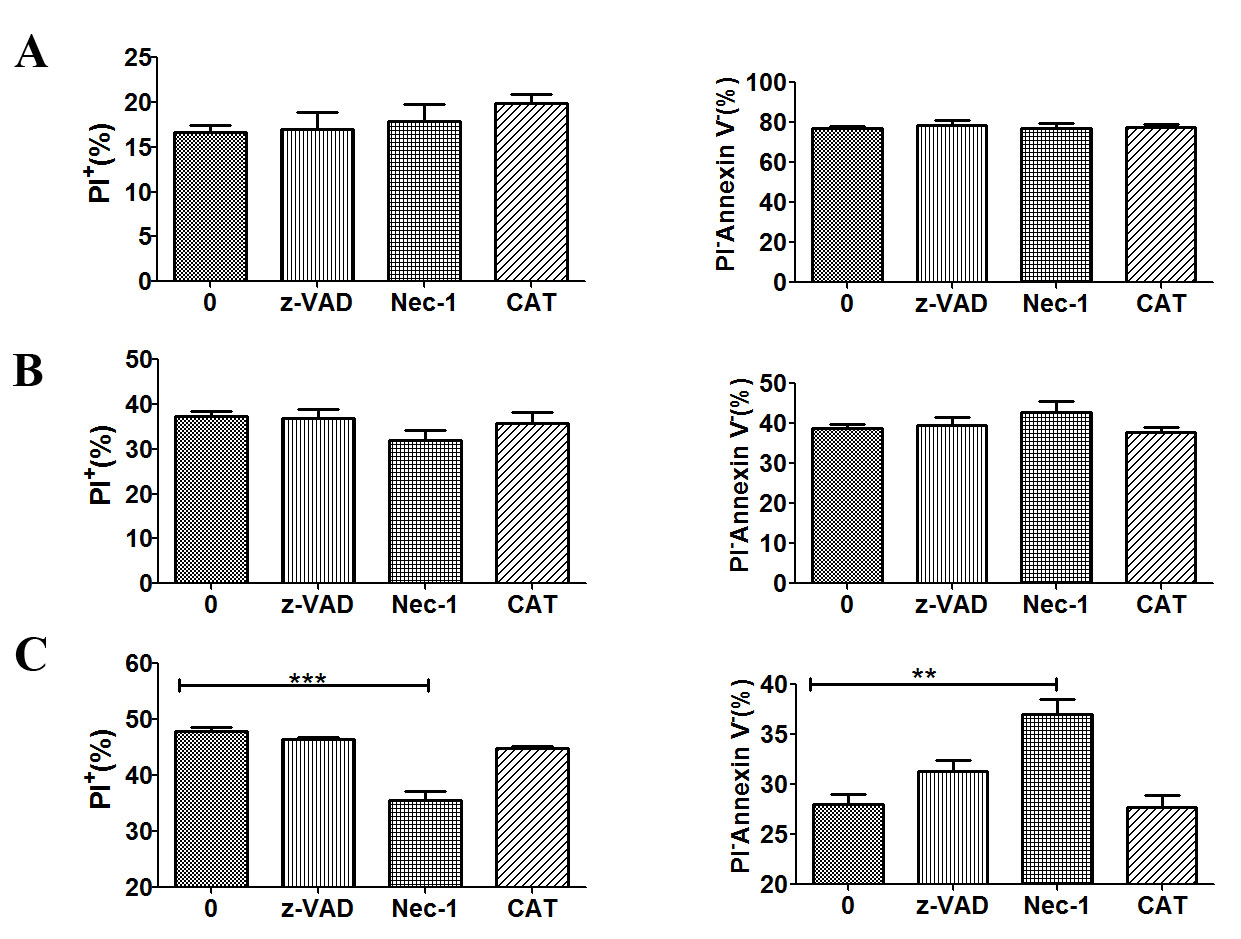


Figure S5. Ripk1 inhibitor reduces the mortality of B cells induces by the joint stimuli. Mouse spleen B cells were exposed to the joint stimuli of R848 and anti-IgM/CD40 after pretreated with the pan-caspase inhibitor zVAD-fmk (z-VAD: 80uM), the necroptosis inhibitor Necrostatin-1 (Nec-1: 60uM) and the ROS inhibitor Catalase (CAT: 200U) for 1 h. The B cells were co-stained with PI and Annexin V-FITC, and cell death was detected by flow cytometry on day2 (A), day 3(B) and day 4(C).
